# Diagnostic Performance of Various Methodologies for Group B S*treptococcus* Screening in Pregnant Woman in China

**DOI:** 10.3389/fcimb.2021.651968

**Published:** 2021-05-24

**Authors:** Kankan Gao, Qiulian Deng, Lianfen Huang, Chien-Yi Chang, Huamin Zhong, Yongqiang Xie, Xiaoshan Guan, Haiying Liu

**Affiliations:** ^1^ Clinical Laboratory, Guangzhou Women and Children’s Medical Center, Guangzhou Medical University, Guangzhou, China; ^2^ School of Dental Sciences, Faculty of Medical Sciences, Newcastle University, Newcastle upon Tyne, United Kingdom; ^3^ Clinical Laboratory, Guangzhou Brain Hospital, Guangzhou Medical University, Guangzhou, China

**Keywords:** group B streptococcus, pregnant women, methodology, diagnostic performance, standard reference method

## Abstract

Maternal vaginal/rectal colonization of group B streptococcus (GBS) is a main risk for neonatal invasive infection. Efficient determination of GBS colonization in pregnant women is crucial. This study aimed to investigate the prevalence of GBS carriage and evaluate the diagnostic performance of six methodologies for GBS screening conducted in China, including blood agar plate, liquid chromogenic medium, and loop-mediated isothermal amplification (LAMP) without pre-enrichment, chromogenic agar plate with pre-enrichment, and GBS antigen detection without and with pre-enrichment in comparison with the standard reference method (Lim broth-enriched subculture with plating on 5% sheep blood agar). Vaginal/rectal swabs were collected from 1,281 pregnant women at 35–37 weeks of gestation. Of them, 309 were taken in triplicate, one for Lim broth-enriched subculture, one for blood agar plate, and the third for GBS antigen detection (Reagent W); 177 were acquired in duplicate, one for Lim broth-enriched subculture and the other for GBS antigen detection (Reagent H); 502 were obtained in duplicate, one for Lim broth-enriched subculture and the other for liquid chromogenic medium; 158 were collected in duplicate, one for Lim broth-enriched subculture and the other for LAMP; and 135 were inoculated in Lim broth-enriched for GBS antigen detection (Reagent W) and subculture with chromogenic agar plate and 5% blood agar plate. The overall prevalence of GBS carriage was 10.1% (130/1,281, 95% CI: 8.5–12.1%) according to the standard reference method. Compared with the standard reference method, the LAMP had excellent performance of sensitivity (100%, 95%CI: 83.4–100%), specificity (94%, 95%CI: 88.1–97.1%), and Yoden index (0.940); as well as the blood agar plate with sensitivity (81.5%, 95%CI: 61.3–93.0%), specificity (100%, 95%CI: 98.3–100.0%), and Yoden index (0.815). The other four methods were not sufficient to reach the threshold in terms of sensitivity or specificity compared to the standard reference method. Furthermore, for LAMP, results can be obtained within 0.5–1 h, while for blood agar plate, which needed 24–48 h, and further identification was required. Our data suggested that the performance of LAMP was highly comparable to the standard Lim broth-enriched subculture and LAMP is considered as an alternative for fast and accurate GBS screening.

## Introduction

*Streptococcus agalactiae*, also known as group B *Streptococcus* (GBS), is a commensal Gram-positive bacterium that can transiently colonize the vagina, lower gastrointestinal tract, and urethra, and is a significant cause of perinatal and neonatal infections worldwide (CDC, 2010; Shabayek and Spellerberg, 2018; Ali et al., 2020; Ding et al., 2020; Plainvert et al., 2020; Zhu et al., 2020). Since early-onset GBS disease (age 0–6 days) occurs *via* vertical transmission during childbirth, bacterial screening and intrapartum antimicrobial prophylaxis (IAP) in pregnant women at 35 to 37 weeks have been developed as routine practices, which has significantly reduced the incidence of neonatal GBS infection (Russell et al., 2017; Vornhagen et al., 2017).

The US Centers for Disease Control and Prevention (CDC) has recommended that isolated bacteria from vaginal/rectal swabs are grown in a selective enrichment medium such as Lim broth (Todd-Hewitt broth supplemented with 15 µg/ml nalidixic acid and 10 µg/ml colistin), followed by subculture on sheep blood agar (CDC, 2010). However, the lack of characteristic hemolysis in approximately 5 to 10% of GBS isolates can lead to a false-negative culture result (Nickmans et al., 2012; Salimnia et al., 2019). In addition, it requires a long turnaround time of 48 to 72 h. In our previous study, it was found that 13,604 cases of GBS and 1,142 GBS-associated deaths in infants <90 days of age annually in China (Ji et al., 2019). However, currently there are no standardized guidelines on GBS screening and prevention in China. Some tertiary hospitals adopt the US CDC guidelines in clinical applications, that is, antibiotic prophylaxis (penicillin, ampicillin, or cefazolin) for pregnant women who are screened positive for GBS (Zhu et al., 2017). Considering that the strategy of IAP has not been conducted universally in China, resulting in a large number of pregnant women couldn’t receive adequate prenatal care including routine screening for GBS. Therefore, the vaginal/rectal colonization status of GBS for them is unknown. In these cases, a more rapid screening method is desirable, especially for supporting urgent decision-making regarding administration of antibiotic prophylaxis. At present, due to lack of standardized guidelines, each clinical laboratory decides the GBS screening method by itself, which has led to various methods to be carried out in different clinical laboratories, and their performances need to be further verified.

Therefore, this study was conducted in a large women and children’s medical center to evaluate the time effectiveness and diagnostic performance of the common GBS screening methods carried out in Chinese medical institutions, including blood agar plate, liquid chromogenic medium, and loop-mediated isothermal amplification (LAMP) without pre-enrichment, chromogenic agar plate with pre-enrichment, and GBS antigen detection without and with pre-enrichment.

## Materials and Methods

### Study Design and Participants

**Figure 1:**
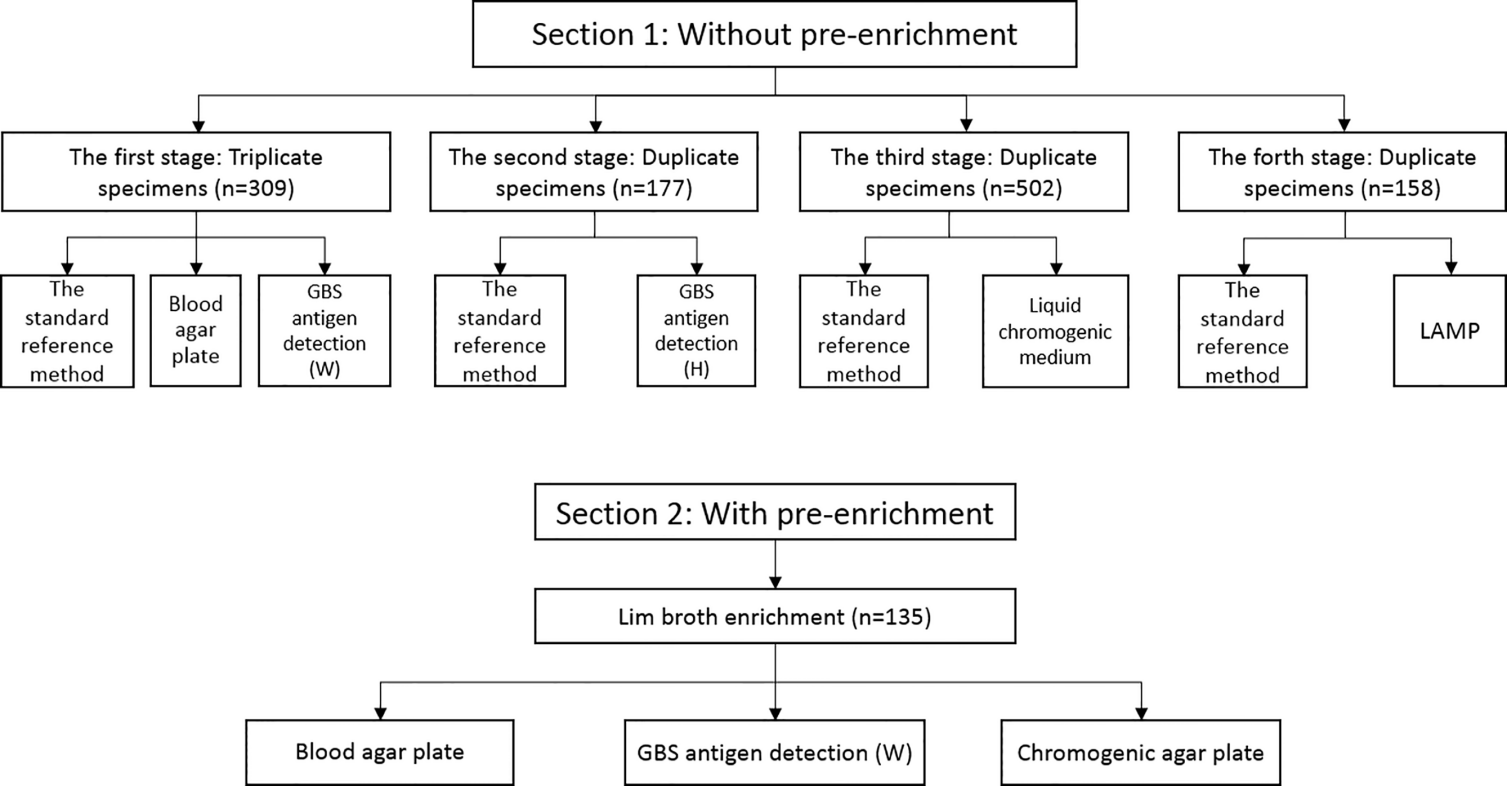
Overall study specimen workflow.

This study was carried out between October 2016 and October 2019. Pregnant women who presented for medical appointments at the obstetric clinic of the Guangzhou Women and Children’s Medical Center, Guangzhou Medical University were recruited. The inclusion criteria were pregnant women at 35–37 weeks of gestation, while the exclusion criterion was any use of antibiotics in the 30 days preceding enrollment. A total of 1,281 participants were recruited. Each participant were swabbed both the lower vagina and the rectum, and all vaginal/rectal specimens were collected on sterile cotton swabs (Kangjian, Taizhou, China). All swabs were firstly inputted into the electronic information system to monitor the transit time once arriving at the laboratory, and then immediately processed in the biosafety cabinet. This study was divided into 2 sections: In the 1st section, four methods without broth pre-enrichment, which consisted of four stages: i) the first stage, 309 participants had swabs taken in triplicate, one for Lim broth-enriched subculture (Dijing, Guangzhou, China), one for blood agar plate (Dijing, Guangzhou, China), and the third for GBS antigen detection (Reagent W, Weimi, Guangzhou, China); ii) the second stage, 177 participants had swabs taken in duplicate, one for Lim broth-enriched subculture and the other for GBS antigen detection (Reagent H, Huaao, Zhuhai, China); iii) the third stage, 502 participants were swabbed in duplicate, one for Lim broth-enriched subculture and the other for liquid chromogenic medium (Dier, Zhuhai, China); iv) the fourth stage, 158 participants had swabs taken in duplicate, one for Lim broth-enriched subculture and the other for LAMP (HiberGene Diagnostics, Dublin, Ireland). The methods in each stage were conducted in parallel with the standard reference method. In the 2nd section, two methods were conducted with broth pre-enrichment. In this section, 135 single swabs were collected, and single swab was inoculated into Lim broth for i) post-enrichment GBS antigen detection (Reagent W, Weimi, Guangzhou, China), ii) subculture on chromogenic agar plate (Dijing, Guangzhou, China), and iii) blood agar plate (Dijing, Guangzhou, China). Two methods in the 2nd section were conducted in parallel with the standard reference method. **Figure 1** outlines the overall study specimen workflow. All specimens were transported to the laboratory of bacteriology research within 4 h.

### Lim Broth Enrichment Subculture and Identification of GBS

As a standard reference in this study, swabs were inoculated into Lim broth for 16 to 24 h at 37°C and subcultured to 5% sheep blood agar plate at 37°C for up to 48 h. The identification was confirmed using a Gram stain, catalase, hippurate hydrolysis (Beisuo, Zhuhai, China), and a commercial automatic bacterial identification system (VITEK 2 COMPACT, bioMérieux, Marcy L’Étoile, France) (Rosa-Fraile and Spellerberg, 2017). It was verified by MALDI-TOF MS (Microflex LT, Bruker Daltonics, Bremen, Germany) when the probability is less than 95%.

### GBS Antigen Detection

The assay was based on colloidal gold immunochromatography technology. The operation was carried out according to the following instructions. The swab specimen in a tube was treated by adding five drops of the extracting solution and squeezed repeatedly. After standing for 5 min, the swab was discarded and two drops of the reaction solution obtained in the above step were added to the reaction strip, which contains nitrocellulose film, polyester film, glass fiber, absorbent paper, polyvinyl chloride board and colloidal gold. The result was visually determined while standing at room temperature for 10–15 min. A positive specimen is defined as a red band occurs on both the test line and the quality control line. Every test involved one negative control and one positive control. In this study, two antigen detection assays, including their extracting solutions and reaction strips, were acquired from two different manufacturers (Weimi and Huaao, China, distinguished by Reagent W and Reagent H, respectively).

### Liquid Chromogenic Medium

This GBS culture analysis system (DL-GBS48, Dier, Zhuhai, China) includes liquid culture medium and culture monitoring instrument. The medium is actually a sealed small culture bottle, containing peptone, yeast extract, chromogenic agent, diluent, mineral oil, inorganic salt, and deionized water. Swab sample was inoculated into the medium; subsequently, the medium was placed in the matched culture monitoring instrument. In anaerobic conditions, GBS can produce an orange-red carotenoid pigment to make the medium into orange, which is recognized by the culture monitoring instrument.

### Loop-Mediated Isothermal Amplification (LAMP)

The LAMP technology has been described previously (Nagamine et al., 2002; McKenna et al., 2017). Shortly, six primers for eight gene regions are designed to generate self-priming dumbbell-shaped templates after isothermal incubation with strand displacement polymerase. Large amounts of amplicon are rapidly produced and this amplification is visualized in real-time on the HiberGene Swift instrument by means of an intercalating fluorescent dye. The operation was performed as follows: The swab specimen was immersed in the 80 ul elution buffer, and the eluate was subjected to enzymatic lysis for 20 min at room temperature. The lysate was then heated to 105°C for 5 min to allow genome DNA denaturation. Then, 25 ml of the denatured lysate is added to the HiberGene reaction strip, which contains primers both for a highly conserved gene region of GBS and an exogenous bacteriophage sequence used as the assay Extraction Control, together with an intercalating dye. Primers, polymerase, intercalating dye for detection of amplified product, and all other reactants, are provided in the HiberGene LAMP reaction kits (HiberGene Diagnostics, Dublin, Ireland).

### Chromogenic Agar Plate

Swabs were inoculated into Lim broth for 16 to 24 h at 37°C and subcultured on chromogenic agar plate (Dijing, Guangzhou, China) at 37°C for up to 48 h. Purple pigment colonies indicate of the presence of GBS.

### Statistical Analysis

For the purposes of clinical validation, the standard reference method was taken by Lim broth-enriched subculture with plating on 5% sheep blood agar. Sensitivity, specificity, positive predictive value (PPV), negative predictive value (NPV), positive likelihood ratio (+LR), negative likelihood ratio (−LR), coincidence rate, and Yoden index for each test methods were evaluated. Furthermore, the level of agreement between each test methods and standard reference method was assessed using the kappa coefficient (κ).

Statistical analysis was performed by SPSS Statistics version 22 (IBM, Chicago, IL, USA). The performance characteristics, including sensitivity and specificity were calculated by 2  ×  2 contingency table for dichotomous outcome of presence or absence of GBS colonization. Confidence intervals for proportions were calculated by using the exact binomial confidence intervals. Consistency analysis (κ) was performed by Kappa test.

## Results

### The Colonization Rate of GBS in Late Pregnant Women

In the 1st section, i) the first stage, among the 309 participants, 27 (8.74%) were positive by the standard reference method; ii) the second stage, of the 177 participants, 23 (13.0%) were positive by the standard reference method; iii) the third stage, out of 502 participants, 38 (7.57%) were positive by the standard reference method; iv) the fourth stage, of the 158 participants, 25 (15.82%) were positive by the standard reference method. In the 2nd section, a total of 135 late pregnant women were enrolled, and 17 (12.6%) were confirmed to be GBS colonized by the standard reference method. The average vaginal/rectal colonization rate of GBS in late pregnancy was 10.1% (130/1,281) with 95% confidence interval: 8.5–12.1%.

## The 1st Section: Performance of Assays Without Broth Pre-Enrichment

### Blood Agar Plate

A total of 309 participants, 22 (7.1%) were GBS positive by direct blood agar culture. Among the 27 Lim broth enrichment positive specimens, five, however, were negative by direct blood agar plate culture, resulting in an overall sensitivity of 81.5% (22/27).

### GBS Antigen Detection

309 and 177 participants were found to be positive of 35 (11.3%) and 45 (25.4%) by GBS antigen detection (reagents W and H, respectively). Unexpectedly, compared to the 27 and 23 positive specimens of the standard reference method, just five were validated by reagents W and H, respectively, displaying low sensitivities of 18.5% (5/27) and 21.7% (5/23), respectively.

### Liquid Chromogenic Medium

502 participants were recruited, and 36 (7.2%) were positive by liquid chromogenic medium. Among the 38 positive subjects of the standard reference method, yet 11 were identified as negative by liquid chromogenic medium, leading to a moderate sensitivity of 71.1% (27/38).

### LAMP

A total of 158 participants, all the Lim broth enrichment positive subjects were confirmed by LAMP, showing a sensitivity of 100%. Meanwhile, among the 133 negative subjects of the standard reference method, 125 were verified by LAMP with a high specificity of 94% (125/133).

## The 2nd Section: Performance of Assays With Broth Pre-Enrichment

A total of 135 specimens were acquired for Lim broth enrichment. 17 (14.4%) and 22 (18.6%) were positive by GBS antigen detection (W) and chromogenic agar plate, respectively. Among the 17 positive specimens of the standard reference method, 13 and 12 were confirmed by post-enrichment GBS antigen detection (W) and chromogenic agar plate with sensitivities of 76.5% (13/17) and 70.6% (12/17), respectively. Of the 118 negative specimens of the standard reference method, 114 and 108 were verified by post-enrichment GBS antigen detection (A) and chromogenic agar plate with specificities of 96.6% (114/118) and 91.5% (108/118), respectively.

Sensitivity, specificity, PPV, NPV, coincidence rate, Yoden index, and kappa (κ) coefficient for each method above were as shown in **Table 1**.

**Table 1:** Diagnostic performance of assays without and with broth pre-enrichment.

Methods	No.(n)	TP	TN	FP	FN	Turnaround time	Sensitivity (95% CI)	Specificity (95% CI)	PPV	NPV	+LR	-LR	Coincidence rate	Yoden index	Kappa
**Without pre-enrichment**															
Blood agar plate	309	22	282	0	5	24–48 h	81.5% (61.3–93.0)	100% (98.3–100.0)	100%	98.3%	–	0.185	98.4%	0.815	0.889
GBS antigen detection (W)	309	5	252	30	22	0.5–1 h	18.5% (7.0–38.7)	89.4% (85.0–92.6)	14.3%	92.0%	1.745	0.912	83.2%	0.079	0.069
GBS antigen detection (H)	177	5	114	40	18	0.5–1 h	21.7% (8.3–44.2)	74.0% (66.2–80.6)	11.1%	86.4%	0.835	1.058	67.2%	0.042	−0.034
Liquid chromogenic medium	502	27	455	9	11	24–48 h	71.1% (53.9–84.0)	98.1% (96.2–99.1)	75.0%	97.6%	37.42	0.295	94.6%	0.692	0.708
LAMP	158	25	125	8	0	0.5–1 h	100%(83.4–100.0)	94.0% (88.1–97.1)	75.8%	100%	16.67	0	94.9%	0.940	0.832
**With pre-enrichment**															
GBS antigen detection (W)	135	13	114	4	4	24–48 h	76.5% (49.8–92.2)	96.6% (91.0–98.9)	76.5%	96.6%	22.5	0.243	94.1%	0.731	0.731
Chromogenic agar plate	135	12	108	10	5	48–72 h	70.6% (44.0–88.6)	91.5% (84.6–95.6)	54.5%	95.6%	8.306	0.321	88.9%	0.621	0.552

TP, true positive; TN, true negative; FP, false positive; FN, false negative; CI, confidence interval; PPV, positive predictive value; NPV, negative predictive value; +LR, positive likelihood ratio; -LR, negative likelihood ratio.

Sensitivity = [TP/(TP + FN)] × 100%, is the proportion of true positives tests out of all participants with the disease or condition.

Specificity = [TN/(TN + FP)] × 100%, is the proportion of true negative tests out of all participants without the disease or condition.

PPV = [TP/(TP + FP)] × 100%, is the probability that participants with a positive screening test truly have the disease or condition.

NPV = [TN/(TN + FN)] × 100%, is the probability that participants with a negative screening test truly don’t have the disease or condition.

+LR = [Sensitivity/(1 − Specificity)] × 100%, the higher the value, the more likely the participants have the disease or condition.

−LR = [(1 − Sensitivity)/Specificity] × 100%, the lower the value, the more likely the participants have not the disease or condition.

Coincidence rate = [(TP + TN)/(TP + TN + FP + FN)] × 100%, is the proportion of true positives and negative tests out of all participants.

Youden index = Sensitivity + Specificity − 1, is the total ability of the screening test to yield a positive result for a participant with the disease or condition and to obtain a negative result for a participant without the disease or condition. The value ranges from 0 to 1, the higher the value, the better the performance of the screening test.

Kappa test is used to measure the consistency between the two judgments, with the value that ranges from −1 to 1. The higher the value, the better the consistency.

## Discussion

This clinical evaluation study covered six comparison methods for analysis without and with broth pre-enrichment including a total of 1,281 participants. The colonization of GBS in pregnant women is a significant risk of neonatal invasive infection. In western China, Among the 210 mothers who were positive for GBS, 16 of their infants were GBS positive, resulting in a 7.6% vertical transmission rate (Chen et al., 2018). In southern China, the overall incidence of invasive GBS infection was 0.55 per 1000 live births (Guan et al., 2018). Compared with neonatal GBS colonization, higher proportions of mothers with neonatal GBS infection who were not screened for GBS (94.4% versus 63.6%) and were not received IAP (94.4% versus 72.7%) at labor were observed (Wu et al., 2019). Therefore, the rapid and accurate screening of GBS is essential, which is closely related to methodology. In this study the colonization rate was found to be 10.1% that was consistent with the previous reports of 3.7–14.5% in China (Dai et al., 2019; Huang et al., 2019), which was close to Korea (11.6%) (Kim et al., 2018), slightly higher than that in India (7.6%) (Santhanam et al., 2017), but much lower than that in America (21.6%) (Edwards et al., 2019). In addition to geography, ethnicity, socioeconomics, health, and nutritional factors, prevalence of GBS colonization in pregnant women is also influenced by sampling and screening methodologies. The isolation and identification of vaginal/rectal swab GBS by post-enrichment subculture is considered the gold standard for GBS screening recommend by US CDC guidelines (CDC, 2010) and European consensus (Di Renzo et al., 2015). Currently, direct blood agar plate culture was performed for GBS screening in most Chinese clinical laboratories. Here, the positive rate of this assay (7.1%) was highly in agreement with study (7.1%) reported by Lu et al. (2014). This assay has the advantage of saving time and reducing costs because of eliminating the pre-enrichment step. However, it should be emphasized that the false negative rate was as high as 18.5%, and this finding was supported by other studies with the false negative rates of 20 to 50% (Philipson et al., 1995; Platt et al., 1995; CDC, 2010; El Aila et al., 2010; Di Renzo et al., 2015).

For GBS antigen detection method, we evaluated two reagents without pre-enrichment. Both of them have observably low sensitivity, PPV, coincidence rate, Yoden index, and Kappa value. However, the sensitivity increased sharply from 18.5 to 76.5% by Lim broth pre-enrichment, still significantly lower than that of 93.1% reported by Matsui et al. (2013) and 93.1% by Takayama et al. (2017). This different capacity may depend to the commercial reagent manufacturers. Although it fails to meet the 90% sensitivity threshold recommended by CDC guidelines. It is still worth mentioning that due to the extremely high values of +LR (22.5) and specificity (96.6%), the positive results of post-enrichment GBS antigen detection method could have important clinical value for fast determination the presence of GBS colonization. Besides, no special equipment and technology is required, and the results are easy to obtain within 0.5 h at the earliest, this method is particularly suitable for the primary medical institutions with low medical reserves.

Liquid chromogenic medium has high specificity (98.1%), NPV (97.6%), +LR (37.42), and coincidence rate (94.6%). As the post-enrichment GBS antigen detection assay, the positive result is of great significance for determining the colonization of GBS. But it requires special equipment for result reading, relatively low sensitivity (71.1%), and no advantage in turnaround time with 24–48 h, resulting in the application scope is not as wide as post-enrichment GBS antigen assay. Among the false positive samples, a large number of Gram-positive *cocci* were identified frequently, such as *Enterococcus faecalis*, *Streptococcus salivarius*, *Streptococcus mutans*, and *Staphylococcus epidermidis*.

LAMP is a novel *in vitro* nucleic acid amplification technique invented by Notomi in 2000 (Notomi et al., 2000). The LAMP exhibited perfect sensitivity (100%) and specificity (94.0%), reliable values of +LR (16.67) and −LR (0), and high consistency (κ = 0.832) in compared to the standard reference method. In addition, results could be obtained in 0.5 h at the earliest. These data provided an evidence of excellent performance for GBS screening (McKenna et al., 2017; Curry et al., 2018). In present study, eight participants were found to be positive by LAMP but negative by post-enrichment subculture. Unfortunately, all the specimens were not available for further discrepant analysis. In fact, nucleic acid amplification test (NAAT) is a popular area in GBS screening study recently, and many NAAT-based screening methods have been developed. Ellem et al. found that NAAT (BD Max GBS) without the need for enrichment could reach the sensitive of 98.4% and specificity of 100% (Ellem et al., 2017). As well, in the study by Couturier et al., the performance of three NAATs (BD Max GBS, Illumigene GBS, and Quidel AmpliVue GBS) was compared with broth enrichment culture method. Compared to that of the composite standard, the sensitivity of NAATs ranged from 98.5 to 100%, while the culture was 70.6%, and no sample with culture-positive but NAAT-negative result was found (Couturier et al., 2014). Nielsen et al. also found that the sensitivity and specificity of GeneXpert^®^ and GenomEra^®^ PCR assays were 92.2% and 99.6%, and 92% and 96.8%, respectively (Nielsen et al., 2020). NAATs were proved to be more sensitive than the post-enrichment subculture as demonstrated by the identification of GBS that were positive by NAATs assay but negative by the post-enrichment subculture method (Buchan et al., 2015; Shin and Pride, 2019). In conclusion, NAATs offer faster time to result, greater throughput, and potentially greater sensitivity, and they are suggested as an alternative for GBS screening in pregnant women (Rosa-Fraile and Spellerberg, 2017; Guo et al., 2019). But a further disadvantage of this assay is loss of the ability to subculture GBS isolates in positive women for antibiotic susceptibility testing. Developing primers for antibiotic resistant gene detection along with GBS NAATs identification may overcome this disadvantage.

The use of chromogenic medium is also recommended by the CDC and European consensus. In this study, post-enrichment inoculation on chromogenic agar plate had a sensitivity of 70.6%, which was much lower than that of 95–100% by El Aila et al. (2010) and 95.7% by El Shahaway et al. (2019), even 91–95% without Lim broth enrichment reported by El Aila et al. (2010). The detecting reliability of various chromogenic agar plate reportedly depends on the commercial supplier (Church et al., 2017). In the study by El Aila et al., the uses of chromogenic medium are GBSDA (BD) and ChromID™ Strepto B (bioMerieux). In the study by El Shahaway et al., the use of chromogenic medium is StrepBSelect™ (Bio Rad). The European Consensus recommends the chromogenic media including StrepBSelect™ (Bio Rad), ChromID™ Strepto B (bioMerieux), and Brillance GBS (Thermo Scientific) (Di Renzo et al., 2015). Chromogenic media provide an easy but not fast approach for GBS identification, with up to 72 h if pre-enrichment. Besides, to avoid false-positive results, the use of GBS chromogenic media requires confirmation of suspected GBS colonies by using additional test (Rosa-Fraile and Spellerberg, 2017).

In conclusion, this study was designed to evaluate the performance of several common GBS screening methods implemented in Chinese clinical institutions. Among them, LAMP has the advantages of being reliable, simple, and rapid to be considered as an alternative for GBS screening. The blood agar plate without pre-enrichment method has a considerable false negative rate, thus a pre-enrichment step is still recommended. Although the GBS antigen analysis with a pre-enrichment step could greatly improve the performance, its sensitivity is still relatively low compared to the standard reference method. The liquid chromogenic medium without pre-enrichment and chromogenic agar plate with pre-enrichment methods both fail to meet the 90% sensitivity threshold, but the performance of various chromogenic media varies depending on the commercial supplier, maybe culture-proven commercial chromogenic media are recommended. Hence LAMP is considered as an alternative for fast and accurate GBS screening.

## Data Availability Statement

The original contributions presented in the study are included in the article/supplementary material, further inquiries can be directed to the corresponding author.

## Ethics Statement

The study was approved by the review board of Guangzhou Women and Children’s Medical Center, Guangzhou, China. Written informed consent for participation was not required for this study in accordance with the national legislation and the institutional requirements.

## Author Contributions

This study was designed and conceived by HL. The analyses and first draft of the paper were undertaken by KG. Statistical analysis was performed by LH and HZ. Microbiological techniques were carried out by QD and XG. Tables and figures were prepared by YX. C-YC provided critical comments on the draft, and managed subsequent revisions. All authors contributed to the article and approved the submitted version.

## Funding

This work was supported by grants from the Guangzhou Science, Technology and Innovation Commission (201804010447), Guangzhou Municipal Health Commission (20201A011024), and Hospital Fund of Guangzhou Women and Children’s Medical Center (YIP-2019-051).

## Conflict of Interest

The authors declare that the research was conducted in the absence of any commercial or financial relationships that could be construed as a potential conflict of interest.
